# Complication of Hepatic Hydatid Cyst Surgery Presenting as Obstructive Jaundice

**DOI:** 10.7759/cureus.35410

**Published:** 2023-02-24

**Authors:** Priya Ahire, Nandhini Iyer, Parth B Gada

**Affiliations:** 1 General Surgery, Grant Government Medical College and Sir JJ Group of Hospitals, Mumbai, IND

**Keywords:** endoscopic management, hydatid cyst surgery, emergency gastroenterology and endoscopy, echinococcus granulosis, post operative complication, endoscopic retrograde cholangiopancreatography (ercp), minimally invasive laparoscopy, surgical obstructive jaundice, hepatic echinococcosis, hydatid cyst of liver

## Abstract

The liver is the commonest organ affected by hydatid disease. We report a rare case of a 25-year-old female patient who was treated surgically for hepatic echinococcosis two weeks ago with laparoscopic excision of hepatic hydatid cyst with marsupialization and omentoplasty. She then presented with features of obstructive jaundice, which is a known complication following hydatid endocystectomy. Cholangiogram revealed a communication of the residual hydatid cyst with right segmental intrahepatic biliary radicals. She was treated with endoscopic retrograde cholangiopancreatography (ERCP)-guided stenting. ERCP is regarded as an important therapeutic strategy for hydatid cysts occurring in the extra biliary tree either as primary or as complications of liver cysts. It facilitates the clearing of hydatid debris from the biliary tree, and the closure of fistulas and bile leaks followed by laparoscopic cholecystectomy when the hydatid cysts are also located in the gallbladder.

## Introduction

Hydatid disease or echinococcosis, an endemic disease of the Mediterranean region is a zoonosis caused by Echinococcus granulosus larvae in areas where there is a close link between sheep, dogs, and humans [1]. Almost 60-75% of cases of Echinococcosis present as liver disease and lead to a wide range of complications, of which cysto-biliary communications remain the most commonly encountered one [2,3].

The occurrence of obstructive jaundice and cholangitis due to hydatid remnants in the biliary tract and the usage of endoscopic methods to manage such complications in the postoperative period is reported in our case.

## Case presentation

**Figure 1:**
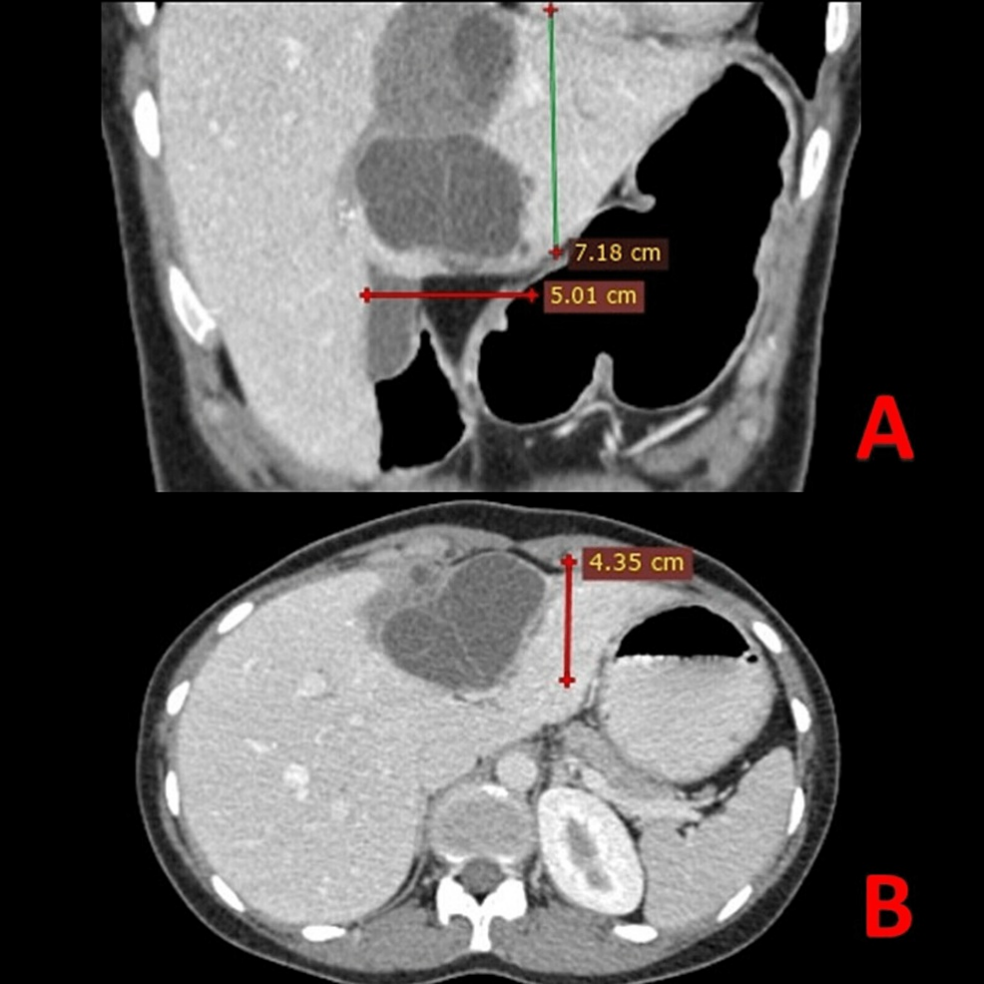
Venous phase of contrast-enhanced CT scan (CECT) (A) Coronal plane, and (B) Axial plane, showing a multi-cystic 7 x 5 x 4.3cm lesion in the segment IVa and IVb of the left lobe of the liver suggestive of hydatid cyst.

We report a rare case of a 25-year-old female patient who presented with obstructive jaundice. She complained of pain in the right upper quadrant of the abdomen, vomiting, and fever with chills. She was treated surgically for hepatic echinococcosis, a multi-cystic lesion of size 7 x 5 x 4.3cm in the segment IVa and IVb of the left lobe of the liver (Figure 1A, 1B) two weeks ago with laparoscopic excision of hepatic hydatid cyst with marsupialization and omentoplasty.

**Figure 2:**
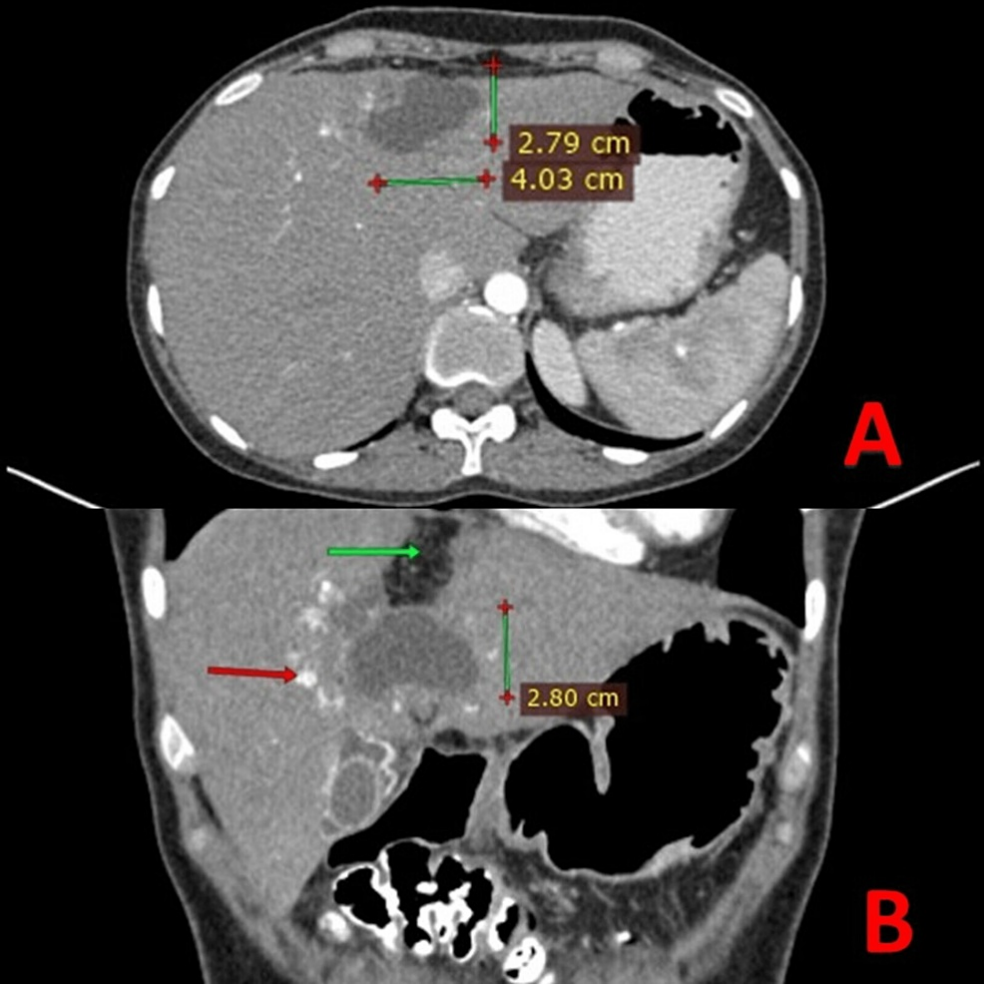
Postoperative contrast-enhanced CT scan (CECT) (A) Axial plane-Arterial phase showing residual cystic lesion in segment IV-a of the liver. (B) Coronal plane showing peripheral foci of calcifications (red arrow) and postoperative omental packing (green arrow).

A panel of blood investigations was done that revealed serum glutamic oxaloacetic transaminase (SGOT) 63 U/l, serum glutamic pyruvic transaminase (SGPT) 87 U/l, total bilirubin 4.5 mg% (direct 3.5 mg% and indirect 1.0 mg%), and alkaline phosphatase 486 U/l. Abdominal sonography was done that revealed a residual cyst in the left lobe of the liver. The contrast-enhanced CT scan (Figure 2A, 2B) showed mild hepatomegaly with a residual cystic lesion in segment IV-a showing peripheral foci of calcifications and postoperative omental packing. These findings collectively pointed to the diagnosis of obstructive jaundice following hydatid endocystectomy.

**Figure 3:**
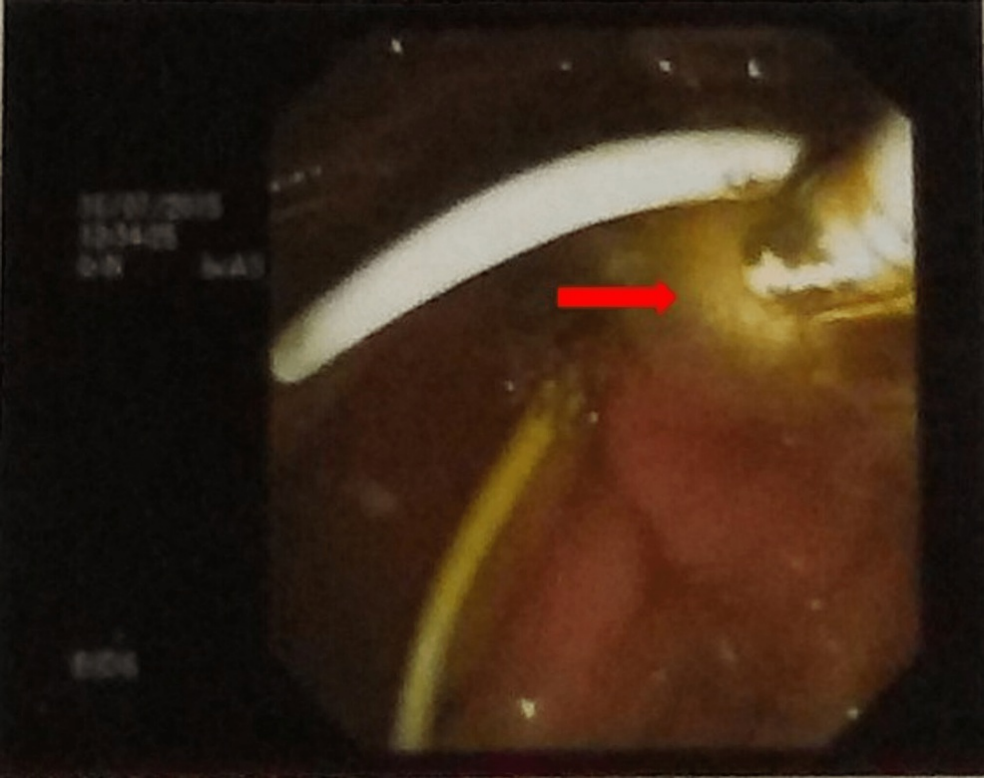
Endoscopic retrograde cholangiopancreatography (ERCP) image White pus-like material retrieved after biliary sphincterotomy and balloon sweep of the common bile duct was done.

Endoscopic retrograde cholangiopancreatography (ERCP) was planned. Cholangiogram revealed normal common bile duct (CBD) with few filling defects. Biliary sphincterotomy and balloon sweep of the CBD were done and white pus-like material was retrieved (Figure 3).

**Figure 4:**
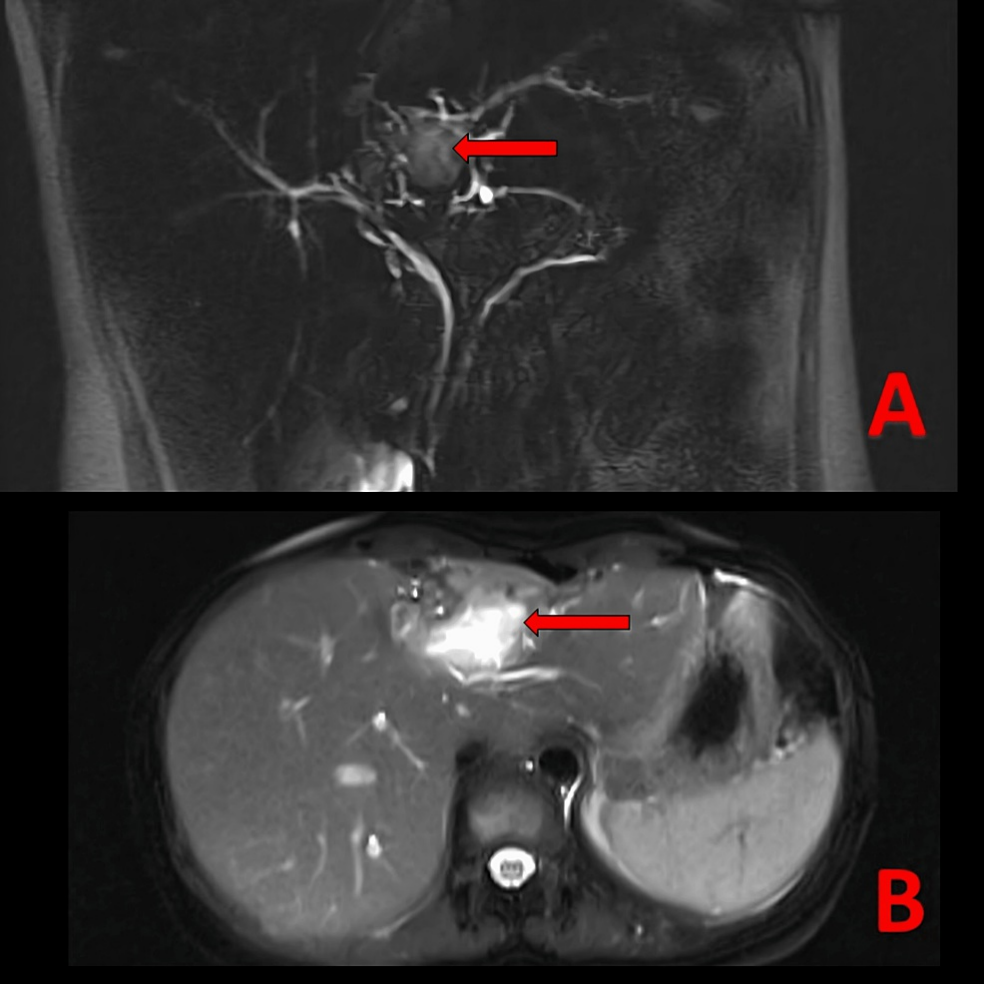
T2-weighted magnetic resonance imaging (MRI) post endoscopic retrograde cholangiopancreatography (ERCP) stenting (A) Coronal plane and (B) Axial plane, showing one of the biliary radicals of the left lobe reaching up to the posterior-superior wall of the residual hydatid cyst with suspicious communication.

7 Fr and prophylactic 5 Fr pigtail stents were placed in the common bile duct and the main pancreatic duct respectively. Magnetic resonance imaging (MRI) post ERCP stenting (Figure 4A, 4B) showed one of the biliary radicals of the left lobe reaching up to the posterior-superior wall of the residual hydatid cyst with suspicious communication. The main pancreatic duct was dilated measuring 3 mm. A well-defined thick-walled cystic lesion 4.8 x 2.8 x 3 cm was visualized in the left lobe of the liver extending up to the subcapsular region.

**Figure 5:**
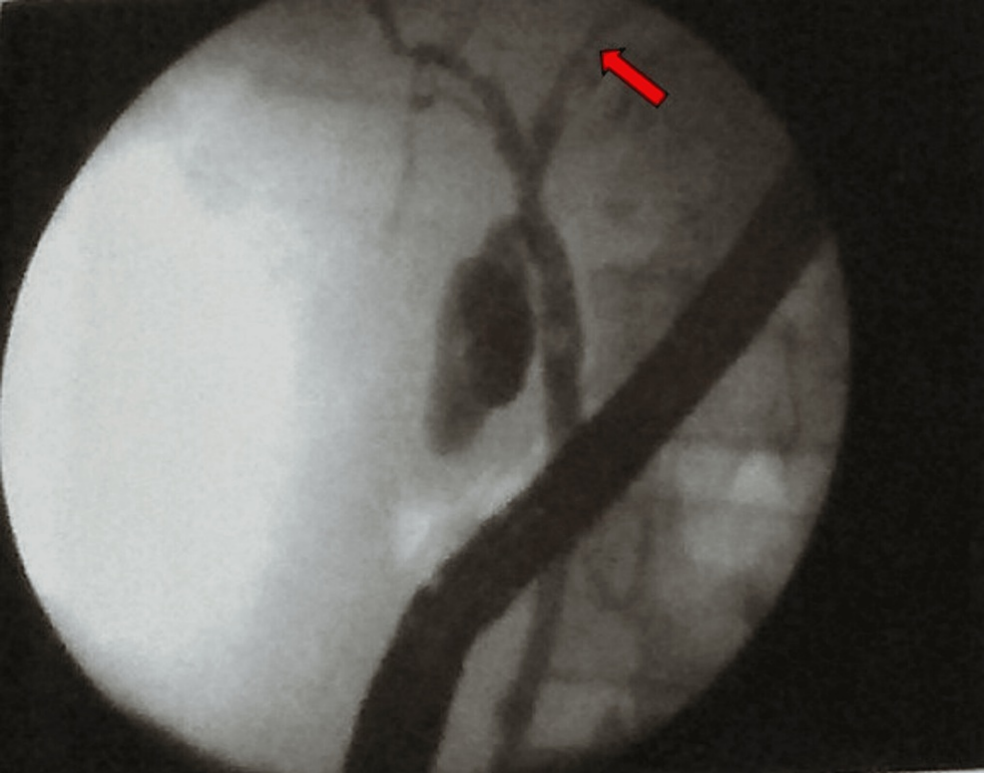
Repeat endoscopic retrograde cholangiopancreatography (ERCP) - Cholangiogram Cholangiogram revealed a free leak of contrast from the left main hepatic duct.

Two weeks post stent placement, a non-enhanced CT scan (NECT) was done, and under CT guidance and aseptic precautions, a Chiba needle was inserted into the residual cystic lesion in segment IVa of the liver. 5ml of non-ionic dye was injected through the needle into the cyst. The post-injection scan showed communication of the cyst with right segmental intrahepatic biliary radicals with the passage of contrast into the duodenum. ERCP was done again and the previously placed stent was removed. Cholangiogram revealed a free leak of contrast from the left main hepatic duct (Figure 5).

A basket trawl was done and stones were retrieved. Another 7 Fr stent was inserted in the left main hepatic duct and free bile drainage was confirmed. The patient thereafter showed a drop in total serum bilirubin levels to the normal range and resolution of jaundice. Subsequent tests and examination over a period of six months of follow-up were normal and the patient remained asymptomatic.

## Discussion

Hydatid disease is a parasitic tapeworm disease caused by the larval stage of Echinococcus granulosus or Echinococcus multilocularis and is endemic mostly in sheep-farming countries [1,4,5]. Liver is the most frequently involved organ followed by the lungs and rarely the spleen, kidneys, bones, and the brain [6].

Complications of hepatic hydatid cysts include rupture into the bile ducts or abdominal cavity, invasion of other abdominal structures, superadded bacterial infections, and mass effect-related complications [3,7]. The onset of fever with chills, nausea, malaise, vomiting, jaundice, and raised hepatic markers should point toward possible complications [3] which was the classic presentation in our patient. Intra-biliary rupture occurs more commonly in the right lobe of the liver (55-60%) [7] followed by the left lobe and biliary ducts (30-35%) and rarely occurs in the common bile duct. It is the most common complication of hepatic hydatids which leads to the formation of a connection between the cyst and the biliary tract which allows the pieces of the germinal layer and daughter vesicles to migrate [8].

Occurrence of acute cholangitis, jaundice, pain, and/or persistent biliary fistula after surgery should raise suspicion for intra-biliary rupture as a possible diagnosis. The clinical features of the patient depend mainly on the size of the communication between the cyst and the biliary tree and can range from asymptomatic to jaundice, cholangitis, cholecystitis, pancreatitis, liver abscess, or septicemia while ruptures which are not diagnosed can directly lead to leakage from the biliary system, formation of a biloma, infection of the cavity and obstructive jaundice after hepatic hydatid cyst surgery [7,9].

There are two forms of intra-biliary rupture of a hepatic hydatid cyst. The first one is an occult rupture which may be asymptomatic and they are identified only during surgery or they may remain undiagnosed. Our patient had an occult rupture in which the fluid from the cyst drains into the biliary tree and the entry of hydatid material can lead to a wide range of biliary complications like hydatid biliary lithiasis, cholangitis and sclerosis odditis which remained undetected during the surgery and presented directly in the postoperative period. Diagnosis of this complication can usually be made by using ultrasound and abdominal CT scan showing dilated common bile duct, jaundice in addition to a cystic lesion in the liver and bile duct dilatation [1,6,7]. The second form is the symptomatic frank intra-biliary rupture which requires prompt timely treatment on detection either with endoscopic procedures or surgery [7].

The choice of treatment is made based on the size of the communication, the location of the cyst, and the experience of the hepatobiliary surgeon [10].

According to our patient’s history and medical records, she had an uncomplicated laparoscopic liver hydatid cyst excision two weeks earlier during which no communication between the hydatid cyst and biliary tree was detected. Liver function tests were normal at the time of discharge. Rupture of the hepatic hydatid cysts into the biliary tree occurs in almost one-fourth of the cases [7], due to high pressures in the cysts reaching up to 80cm of H2O leading to obstructive jaundice and cholangitis. The possible explanations for the occurrence of obstructive jaundice could be either intra-biliary rupture of hepatic hydatid cyst or primary hydatid cyst of the extrahepatic duct, the former being more likely in our case based on the imaging findings.

The large size of the cyst (>7.5 cm), location in the central segments of the liver, and the advanced stages of the disease are the three main risk factors for intra-biliary rupture [3,11]. Our patient had all these risk factors with the size being 7 x 5 x 4.3 cm multi-cystic lesion located in segments IVA and IVB in the left lobe of the liver with calcific foci in the wall suggestive of the disease being in the advanced stages. An increase in the intra-cystic pressure can lead to compression of the adjacent bile duct walls leading to necrosis. Small biliary ducts may be incorporated within the pericyst eventually causing the biliary ducts to rupture. If only small fissures are present, patients may be asymptomatic however if frank communications are present or perforation has occurred, patients present with obstructive jaundice and cholangitis which was the typical presentation in our patient.

In recent times the diagnosis and treatment of complications during the pre- and post-operative periods heavily rely on minimally invasive methods. For patients with adverse biliary events postoperatively, endoscopic retrograde cholangiopancreatography (ERCP) is indicated. Clinical clues pointing towards obstruction such as jaundice accompanied by biochemical markers suggestive of cholestasis, and a radiological picture showing a dilated biliary duct system or evidence of hydatid elements in the bile ducts all suggest possible preoperative frank rupture and require ERCP. Although ERCP is a highly sensitive test (86-100%) [3], its routine use preoperatively with prophylactic endoscopic sphincterotomy (ES) in cases where a minor cysto-biliary communication is suspected still remains controversial. This debate is fuelled furthermore by the increasing use of magnetic resonance cholangiopancreatography (MRCP). ERCP shows linear wavy filling defects of laminated hydatid membrane into the common bile duct, duodenum, or projecting from the ampulla of Vater and may introduce a catheter from the biliary ducts into the hepatic hydatid cyst to diagnose the communication [3,12].

As done in our patient, a sphincterotomy to remove the cysts and the membranes with the help of a basket or an occlusion balloon is often needed. ERCP is regarded as an important therapeutic strategy for hydatid cysts occurring in the extra biliary tree either as primary or as complications of liver cysts. It facilitates the clearing of hydatid debris from the biliary tree, and the closure of fistulas and bile leaks followed by laparoscopic cholecystectomy when the hydatid cysts are also located in the gallbladder. Moreover, complicated hydatid cysts require preoperative MRCP for diagnosis so that the treatment modality is not delayed [7]. Continuously draining biliary fistulae are defined by a high amount of biliary drainage lasting more than 10 days postoperatively and ERCP with stenting is advised in these cases. Although the risk of biliary leakage is higher in the earlier parts of the postoperative period, this risk begins to decrease from the tenth day after the surgery [2].

We conducted an extensive literature search, comparing different approaches used to treat or prevent the occurrence of biliary fistula in hepatic echinococcosis (Table 1).

**Table 1:** Results of our literature search Comparison of different approaches used to treat or prevent the occurrence of biliary fistulas in hepatic echinococcosis. ERCP: Endoscopic retrograde cholangiopancreatography

Author	Approach and Results
Ozturk et al., 2009 [12] - 13 patients	ERCP is an important intervention as it shows structural changes of the external biliary tract and endoscopic sphincterotomy has a limited effect on these changes. Stents were used in selected cases.
Rodriguez et al., 1998 [13] - 25 patients	Endoscopic sphincterotomy - fistula closed in 25 days.
Tekant et al., 1996 [14] - 10 patients	9/10 patients treated by endoscopic sphincterotomy resolved in seven days.
Adas et al., 2010 [15] - 109 patients all treated by ERCP	70 patients - endoscopic sphincterotomy, only 22 patients - 10F biliary stent insertion, 17 patients - 7F biliary stent insertion; conclusion - stent placement advocated in high output fistulas and 10F stent considered most superior with best results.
Cicek et al., 2007 [2] - 41 preoperative and 69 postoperative subjects	Nasobiliary drainage catheter along with endoscopic sphincterotomy and extraction of hydatid cyst remnants for patients who had filling defects on cholangiogram disadvantage-lengthy hospital stay.
El-Gendi et al., 2018 [16] - 54 patients	Prophylactic endoscopic sphincterotomy (ES) provides a significant reduction in postoperative bile leak rate with shorter hospital stay after partial cystectomy of hydatid cyst. Biliary fistula in patients with ES has significantly lower daily output with a shorter time of drain removal and shorter time to closure than patients without ES.
Atahan et al., 2011 [17] - 68 patients	Gamma-glutamyl transferase (GGT) as a useful laboratory test for predicting suspicious cysto-biliary communications beforehand. If such a communication is found, the biliary system opening should be sutured with absorbable material which may or may not be accompanied by drainage of the cystic duct. If there is no opening found on the biliary tract, preoperative factors must be assessed. If they predict a possible communication, cystic duct should be drained.
Muhammedoğlu et al., 2021 [18] - 122 patients	ERCP as the chief method for the diagnosis and management of ruptured hepatic hydatid cysts. If the rupture into the bile ducts is high flowing or a biliary fistula is detected which has a continuous flow, they can be drained non-surgically with interventions like ERCP and endoscopic sphincterotomy, biliary stenting and drainage using nasobiliary catheter.
Trigui et al., 2021 [19] - 54 patients	The treatment of fistulas was based on DITFO (internal trans-fistula drainage) technique.
El Nakeeb et al., 2017 [20] - 123 patients	Intraoperative suturing and the T tube insertion led to complete healing of cystobiliary fistula, and postoperative ERCP and tubal drainage led to a rapid reduction in the bile output and the healing of the fistulas after 9±2.6 days.

Thus, ERCP should be performed at the earliest, when there is any evidence of bile leak in the postoperative period because delays may lead to infective complications.

## Conclusions

Without timely diagnosis and management, complications of liver echinococcosis which occur in about one-third of patients may be life-threatening. Presentation of an operated case of hydatid cyst of the liver with features of biliary tract involvement like obstructive jaundice and cholangitis suggests possible complication of intra-biliary rupture, which is a rare case and requires early diagnosis and prompt intervention with ERCP for better prognosis of the disease. It is imperative to have adequate knowledge of diagnostic imaging clues to guide the overall management strategy.
